# Improvement of Sympathovagal Balance by Regular Exercise May Counteract the Ageing Process. A Study by the Analysis of QT Variability

**DOI:** 10.3389/fphys.2022.880250

**Published:** 2022-04-20

**Authors:** Beatrice De Maria, Daniela Lucini, Mariana de Oliveira Gois, Aparecida Maria Catai, Francesca Perego, Mara Malacarne, Massimo Pagani, Alberto Porta, Laura Adelaide Dalla Vecchia

**Affiliations:** ^1^ IRCCS Istituti Clinici Scientifici Maugeri, Milan, Italy; ^2^ BIOMETRA Department, University of Milan, Milan, Italy; ^3^ Exercise Medicine Unit, IRCCS, Istituto Auxologico Italiano, Milan, Italy; ^4^ Department of Physical Therapy, Federal University of São Carlos, São Carlos, Brazil; ^5^ Department of Biomedical Sciences for Health, University of Milan, Milan, Italy; ^6^ Department of Cardiothoracic, Vascular Anesthesia and Intensive Care, Policlinico San Donato, San Donato Milanese, Milan, Italy

**Keywords:** physical exercise, heart rate variability, QT interval variability, complexity, autonomic nervous system, athletes, half-marathon, ageing

## Abstract

QT interval (QT) variability analysis provides pathophysiological and prognostic information utilized in cardiac and non-cardiac diseases, complementary to those obtained from the analysis of heart period (HP) variability. An increased QT variability has been associated to a higher risk for cardiac events and poorest prognosis. Autonomic cardiovascular adaptation to internal and external challenges, such those occurring in athletes exposed to high levels of physical stress and in ageing could also be deepen by analyzing QT variability, searching for early prognostic signatures. The aim of the study was to analyze the QT variability and cardiac control complexity in a group of middle-aged half-marathon runners at baseline (B) and at a 10-year follow-up (FU). We found that the overall QT variability decreased at FU, despite the inescapable increase in age (52.3 ± 8.0 years at FU). This change was accompanied by an increase of the HP variability complexity without changes of the QT variability complexity. Of notice, over the years, the group of athletes maintained their regular physical activity by switching to a moderate intensity rather than strenuous. In conclusion, regular and moderate exercise over the years was beneficial for this group of athletes, as reflected by the decreased overall QT variability that is known to be associated to lower cardiovascular risk. The concomitant enhanced cardiac control complexity also suggests a trend opposite to what usually occurs with ageing, resulting in a more flexible cardiac control, typical of younger people.

## Introduction

In the last years, more and more attention has been paid to the evaluation of the cardiovascular risk in athletes exposed to very high levels of physical stress, focusing on the medium-long term effects (Albert et al., 2000; Kim et al., 2012; Dalla Vecchia et al., 2014; Lear et al., 2017; Eijsvogels et al., 2018; Dalla Vecchia et al., 2019; De Maria et al., 2021).

A recent study (De Maria et al., 2021) demonstrated that in a group of middle-aged amateur half-marathon runners, considering a 10-year follow-up, the shift from a strenuous exercise training to a still regular but lower level of exercise, namely a moderate one, was associated to a rebalancing of the sympatho-vagal modulation directed to the heart. Such changes were accompanied by a very low occurrence of cardiovascular disease (De Maria et al., 2021). This would represent a key finding, as an enhanced vagal and decreased sympathetic cardiac modulation to the heart is known to be associated with a reduced risk of cardiovascular events (La Rovere et al., 1998; La Rovere et al., 2001; Thayer et al., 2010; Pinna et al., 2017), confirming physical exercise among the effective cardiovascular prevention measures (Thompson et al., 2003). The aforementioned study was based on the analysis of the heart period (HP) variability, without considering the analysis of the spontaneous fluctuations of the QT interval (QT), that has been successfully applied to study the cardiac neural control to provide, together with HP variability analysis, complementary and non-redundant information (De Maria et al., 2019). Indeed, QT variability analysis provides important pathophysiological insights and is useful for risk stratification in many diseases (Baumert et al., 2016). An increased QT variability has been observed in several cardiac, non-cardiac and metabolic diseases, such as coronary artery disease, hypertension, mental disorders and diabetes mellitus (Baumert et al., 2016). An increased QT variability has also been associated to sudden cardiac death (Maison-Blanche and Coumel, 1997; Singh et al., 1997) and adverse prognosis in patients after myocardial infarction (El-Hamad et al., 2020).

The application of QT variability analysis for the study of cardiac control in athletes is still limited (Yanık et al., 2021; Cavarretta et al., 2022). The aim of the present study is to evaluate the complementary information derived by QT variability assessment, including its complexity, with respect to HP variability, in a group of middle-aged half-marathon runners studied over a 10-year follow-up period. The results of the QT variability and complexity analyses will be compared and discussed in relation to previous data derived from the HP variability analysis (De Maria et al., 2021).

Together with the more traditional and widely applied power spectral analysis, the analysis in the information domain, i.e. complexity analysis, provides information about the regularity of the HP and QT variability series. Complexity analyses are based on the estimation of the complexity of HP and QT series *via* the assessment of the information carried by a new sample of the series that cannot be explained by a combination of past samples (Porta et al., 1998a; Porta et al., 2013).

## Materials and Methods

### Population and Experimental Protocol

The experimental protocol has fully been described previously (Dalla Vecchia et al., 2014; De Maria et al., 2021). The study population consisted of a group of 18 half-marathon runners (17 males) first studied at the fifth Corripavia race and then at a 10-year follow-up. For convenience, the data collected firstly will hereinafter be referred to as baseline (B) (Dalla Vecchia et al., 2014), while those collected at follow-up as FU (De Maria et al., 2021). The demographic and clinical features of the enrolled population at FU are shown in Table 1.

**TABLE 1:** Demographic and clinical features of the enrolled population.

Females/Males, *n*	1/17
Age, years	52.3 ± 8.0
BMI, kg/m^2^	23.1 ± 1.7
Height, cm	176 ± 5
Weight, kg	71.3 ± 7.7
Abdominal circumference, cm	87 ± 6.7
Smokers, *n* (%)	2 (11)
Vitamin supplements, *n* (%)	12 (67)
Vegetarians, *n* (%)	3 (17)
Slept hours per night, hours	6.8 ± 0.8
Physical exercise, hours per week	6.5 ± 3.1

BMI, body mass index. Continuous data are presented as mean ± standard deviation, while categorical variables as absolute number (percentage).

Briefly, the athletes were studied during supine resting (REST) and active standing (STAND), both at B and FU. The electrocardiogram (ECG) from a modified lead II (AT-MIO 16E2, National Instrument at B and LAB3, Marazza, Monza Italy at FU) was acquired for 5 min at REST and for 4 min during STAND. Sampling rate was fixed at 300 Hz at B and at 1000 Hz at FU. Participants were asked to avoid caffeinated and alcoholic beverages in the 24 h preceding the test. They were also asked to avoid physical exercise in the 72 h preceding the experimental session (Furlan et al., 1993).

Written informed consent was obtained from all subjects involved in the study. The study was conducted according to the guidelines of the Declaration of Helsinki, and approved by the Ethics Committee of IRCCS Istituti Clinici Scientifici Maugeri (protocol code 2277CE, date of approval 12/03/2019).

### Heart Period and QT Interval Time Series Extraction

HP and QT beat-to-beat time series were derived from the ECG acquired during the protocol. The HP was approximated as the time distance between two consecutive R wave apexes detected on the ECG by an automatic algorithm, while the QT interval was approximated as the temporal distance between the second R peak of the correspondent HP and the end of the following T wave (Porta et al., 2010; De Maria et al., 2019). The end of the T wave was fixed where the absolute value of the first derivative calculated on the T-wave downslope was smaller than 30% of the value of the steepest slope of the T-wave (Porta et al., 2010; De Maria et al., 2019). None of the analyzed signals exhibited biphasic T waves (Porta et al., 1998b).

R wave peak detections were visually checked to avoid misidentification. Correction of the HP and QT time series was implemented in case of ectopic beats, which never exceeded 5% of the series length.

For each subject, stationary segments of 300 consecutive beats were selected for further analysis in each experimental condition, i.e. REST and STAND.

After linear detrending of the selected segments, mean and variance of the HP and QT series were calculated and named as μ_HP_ and σ^2^_HP_, μ_QT_ and σ^2^_QT_, respectively.

### Power Spectral Analysis

Parametric power spectral analysis was performed over the QT and HP series. The series were modelized as an autoregressive model whose order was chosen according to Akaike information criterion in the range from 10 to 16 and the coefficients were estimated *via* least squares method solved *via* the Levinson-Durbin recursion. The series were then decomposed into power spectral components. The sum of the absolute power of the spectral components of QT series whose central frequency dropped in the low frequency band (LF band, 0.04–0.15 Hz) was labelled as LF_QT_ and taken as an index of the sympathetic modulation directed to the ventricles (Baumert et al., 2011; Porta et al., 2011; De Maria et al., 2019). The sum of the absolute power of the spectral components of the HP series whose central frequency dropped in the high frequency band (HF band, 0.15–0.4 Hz) was labelled as HF_HP_ and taken as an index of the vagal modulation directed to the sinus node (Malliani et al., 1991). HF_HP_ was expressed in absolute and normalized units (Malliani et al., 1991).

### Heart Period and QT Interval Complexity Analyses

The complexity of the HP and QT series was calculated by the corrected conditional entropy (CCE) method, originally described in (Porta et al., 1998a). Briefly, the conditional entropy is based on the estimation of the complexity of a series *via* the assessment of the information carried by a new sample that cannot be explained by a combination of past samples.

The dynamic of the QT and HP series was quantized into six levels and conditional entropy was assessed over the quantized series. The strategy followed to correct the estimate of conditional entropy and to make robust its estimate was described in (Porta et al., 1998a). The application of a correction allowed us to find a minimum of CCE with respect to the number of past samples utilized to condition the evolution of the current one. This minimum represents the best compromise between the ability of past sample to condition the temporal evolution of the series by favoring the reduction of future uncertainty and the natural tendency of conditional probability to decrease towards 0 when the length of the conditioning patterns increases, thus leading to the unavoidable decline of the conditional entropy to 0. The minimum of the CCE was taken as complexity index (CI). CI was computed over HP and QT series (CI_HP_ and CI_QT_), respectively. CI ranges from 0 (the current value does not carry information given past samples indicating maximum predictability and null complexity) to the Shannon entropy, representing the maximum information carried by the current value. Normalized CI of HP (NCI_HP_) and QT (NCI_QT_) series were calculated by dividing CI_HP_ and CI_QT_ by the Shannon entropy of HP and QT series, respectively. Both NCI_HP_ and NCI_QT_ range from 0 (null predictability) to 1 (perfect predictability). The higher the CI_HP_, CI_QT_, NCI_HP_ and NCI_QT_, the higher the complexity of the HP and QT series and the lower their predictability (Porta et al., 2013).

### Statistical Analysis

Two-way repeated measure analysis of variance (ANOVA, one factor repetition, Holm-Sidak test for multiple comparison) was applied to test the differences between the two experimental conditions (i.e. REST and STAND) at B and FU.

In both figures and tables, continuous data are presented as mean ± standard deviation, while categorical variables as absolute number (percentage).

All the statistical analyses were performed using the commercial software Sigmaplot (version 11.0, Systat Software, Inc., Chicago, IL, United States). A *p*-value <0.05 was always considered as significant.

## Results

Table 1 summarizes the demographic and clinical features of the studied population. Compared to B, the athletes’ weekly training was significantly lower at FU, from 14.6 ± 2.9 to 6.5 ± 3.1 h per week, as already described in (De Maria et al., 2021).

**FIGURE 1:**
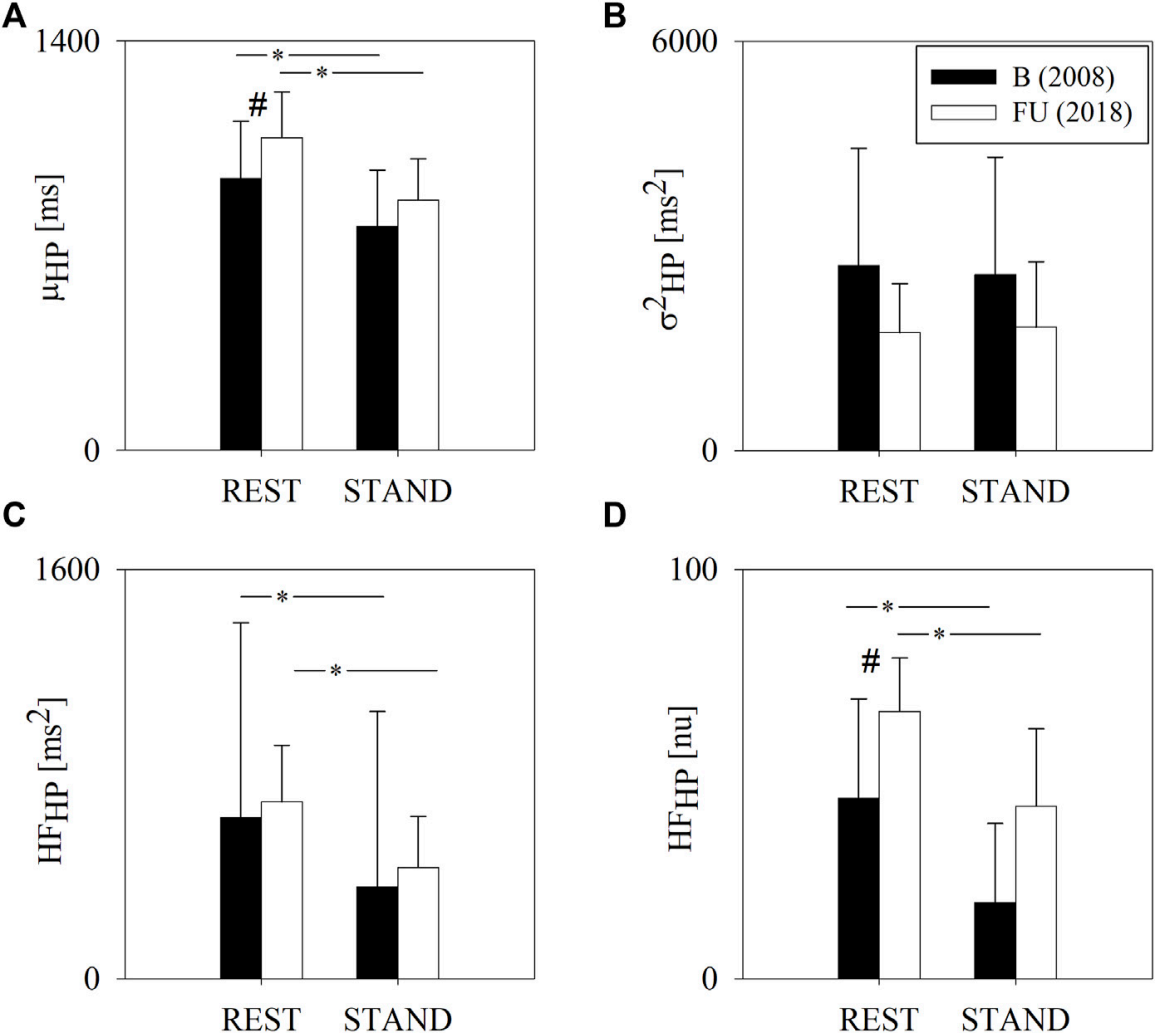
Results of the HP variability analysis in a group of amateur half-marathon runners at baseline (B, black bars) and at a 10-year follow-up (FU, white bars) in resting condition (REST) and during active standing (STAND). The bar graphs show the HP interval mean (μ_HP_, panel **(A)**), HP variance (σ^2^_HP_, panel **(B)**) and the power of HP variability in the high frequency band (HF_HP_ expressed in absolute and normalized units, panels **(C**,**D)**, respectively). Data are presented as mean ± standard deviation. # indicates *p* < 0.05 FU vs B, while *p < 0.05 REST vs STAND.

Figure 1 summarizes the results of the HP variability analysis at B and FU in the group of half-marathon runners, as already presented in (De Maria et al., 2021).

**FIGURE 2:**
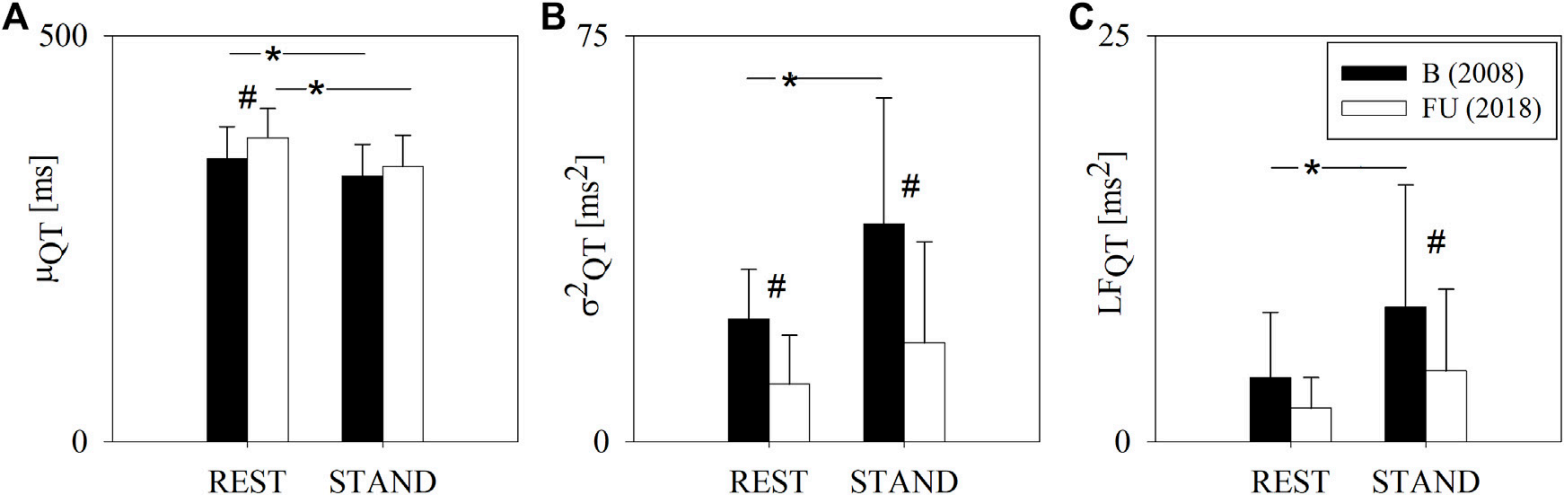
Results of the QT variability analysis in a group of amateur half-marathon runners at baseline (B, black bars) and at a 10-year follow-up (FU, white bars) in resting condition (REST) and during active standing (STAND). The bar graphs show the QT interval mean (μ_QT_, panel **(A)**), QT interval variance (σ^2^_QT_, panel **(B)**) and the absolute power of QT variability in the low frequency band (LF_QT_, panel **(C)**). Data are presented as mean ± standard deviation. # indicates *p* < 0.05 FU vs B, while *p < 0.05 REST vs STAND.

Figure 2 shows the results of the QT variability analysis at B and FU in the two experimental conditions, i.e. REST and STAND. At REST, μ_QT_ was higher at FU compared to B. STAND induced a decrease of μ_QT_ both at B and FU. σ^2^_QT_ was lower at FU compared to B both at REST and during STAND. At FU, from REST to STAND there were no σ^2^_QT_ changes, while at B, STAND induced an increase of σ^2^_QT._ Analogously, LF_QT_ was lower at FU compared to B, both at REST and during STAND, where the difference was significant solely during STAND. LF_QT_ increased significantly from REST to STAND only at B and not at FU.

**FIGURE 3:**
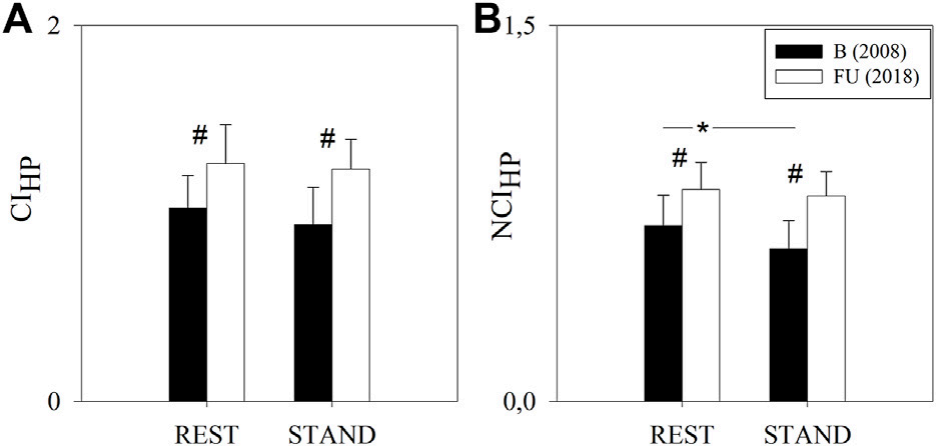
Results of the complexity analysis of the HP series in a group of amateur half-marathon runners at baseline (B, black bars) and at a 10-year follow-up (FU, white bars) in resting condition (REST) and during active standing (STAND). The bar graphs show the complexity index (CI_HP_, panel **(A)**) and the normalized complexity index (NCI_HP_, panel **(B)**). Data are presented as mean ± standard deviation. # indicates *p* < 0.05 FU vs B, while *p < 0.05 REST vs STAND.

Figure 3 shows the results of the complexity analysis over the HP series. Both the CI_HP_ and NCI_HP_ were higher at FU compared to B in both the experimental conditions (i.e. REST and STAND). NCI_HP_ decreased from REST to STAND only at B, while CI_HP_ remained unchanged both at B and FU.

**FIGURE 4:**
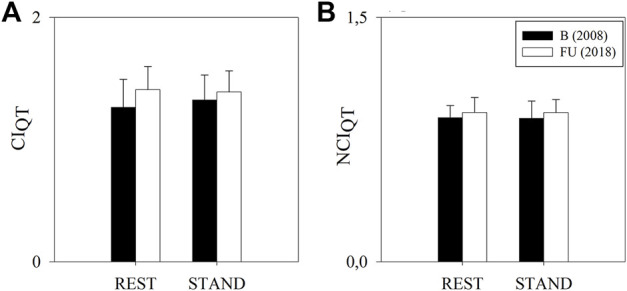
Results of the complexity analysis of the QT series in a group of amateur half-marathon runners at baseline (B, white bars) and at a 10-year follow-up (FU, black bars) in resting condition (REST) and during active standing (STAND). The bar graphs show the complexity index (CI_QT_, panel **(A)**) and the normalized complexity index (NCI_QT_, panel **(B)**). Data are presented as mean ± standard deviation.

Figure 4 shows the results of the complexity analysis over the QT series. No variation was observed for any of the considered indices between FU and B and between REST and STAND.

When the analyses were repeated excluding the only female subject of the study, the above results were confirmed.

## Discussion

The present study was carried out in a group of middle-aged non-elite half-marathon runners within the *Corripavia* protocol (Dalla Vecchia et al., 2014; De Maria et al., 2021). They were evaluated by means of the QT variability and QT complexity analyses at baseline (fifth *Corripavia* race) and at a 10-year follow-up.

At FU, the following findings were identified: *1*) the QT variability decreased; *2*) the complexity of the HP variability increased; *3*) the QT variability complexity did not vary.

### Effects of Regular Physical Exercise on QT Variability

The analysis of the HP variability, utilized in the previous study (De Maria et al., 2021), demonstrated that this same group of athletes exhibited an increased cardiac vagal modulation and a decreased cardiac sympathetic modulation at FU compared to B (Dalla Vecchia et al., 2014; De Maria et al., 2021), the opposite of what usually occurs with ageing (Lakatta and Levy, 2003). These paradoxical findings have been interpreted as the result of the regular physical activity, that in our group of middle-aged athletes was changed over the years in terms of intensity, i.e. from a strenuous to a moderate level of exercise, but has remained regular. The consequent favorable changes in autonomic balance would imply a decreased risk for cardiovascular events, as lower cardiac sympathetic modulation and higher vagal are known to be associated with reduced risk and better outcomes in several pathological conditions, such as heart failure, myocardial infarction, hypertension and diabetes (Liao et al., 1996; Rovere et al., 1998; La Rovere et al., 2001; Thayer et al., 2010; Pinna et al., 2017).

It is recognized that QT variability analysis may furnish additional prognostic indices (Baumert et al., 2016), in particular augmented QT variability has been associated to sudden cardiac death (Maison-Blanche and Coumel, 1997; Singh et al., 1997; Atiga et al., 1998; Haigney et al., 2004), which represents an open issue in both elite and non-elite athletes (Marijon et al., 2015). In the present study, we focused on the analysis of QT variability and cardiac neural control complexity to test the hypothesis that the more balanced HP variability demonstrated in (De Maria et al., 2021) would also be accompanied by a reduction of the overall QT variability. Indeed, the relation between HP and QT variabilities is complex, where only a portion of the QT variability is related to the HP variability (Porta et al., 1998c; El-Hamad et al., 2019), while some changes in the QT variability are independent. The proportion of the QT variability unrelated to HP variability is not negligible, has been proven to be clinically significant (El-Hamad et al., 2019) and is the only portion responsible of the overall increase of QT variability caused by sympathetic enhancement during head-up tilt test (Porta et al., 2010). In addition, decoupling between HP and QT variability has been observed with the ageing process (Baumert et al., 2013).

Our results showed a decreased overall QT variability at FU, both while supine and during active standing, together with a tendency to a diminished LF_QT_ index, a marker of the sympathetic modulation directed to the ventricles (De Maria et al., 2019). These results corroborate those derived from the HP variability analysis (De Maria et al., 2021) and reinforce the concept that a regular and moderate dose of physical exercise is effective in reducing the cardiovascular risk (Shiroma and Lee, 2010; Wen et al., 2011; Dalla Vecchia et al., 2014; Tesema et al., 2019; De Maria et al., 2021).

These findings are further confirmed by those derived from the complexity analysis of the HP variability, as an overall increase of the HP complexity has been observed, independently from the experimental condition. In this group of middle-aged athletes, the augmented HP complexity found at FU is in keeping with a reduced overall sympathetic cardiac modulation and enhanced vagal one (Catai et al., 2014; Milan-Mattos et al., 2018)*.*

The female participant showed similar results as the male ones. This finding is not obviously sufficient to draw any conclusion about sex influences in the response of the cardiovascular neural control to medium and long-term physical training.

### Effect of Regular Physical Exercise on Ageing

Interestingly, the observed increase of HP complexity after at least 10 years of regular exercise might sound paradoxical, as cardiac control complexity is usually reduced by ageing (Kaplan et al., 1991; Catai et al., 2014), as well as by a number of pathological conditions (Goldberger et al., 2002). Therefore, a regular and moderate physical exercise in this group of athletes seems to counteract some effects of ageing on heart control.

The interpretation of QT variability results with respect to ageing is less straightforward. In fact, the effects of ageing processes on the QT variability are still debated (Baumert et al., 2016; Piccirillo et al., 2020), because of the paucity of studies and some contrasting results. However, most studies reported an increased QT and HP variability ratio with ageing, probably dragged by the HP variability decrease more than the QT variability increase (Baumert et al., 2016).

In the athletes of the current study, QT variability decreased at the 10-year follow-up, thus suggesting a rejuvenation of the cardiac neural control. Indeed, in an aged group of athletes regularly exercising, we observed a trend of QT variability opposite to that expected with ageing only. Consistently, they were characterized by a very low cardiovascular disease occurrence (De Maria et al., 2021). An additional confounding factor is the QT-HP relationship that induces a transfer of information from HP to QT and might influence the amount of complexity genuinely attributable to QT dynamics. Future studies should check the impact of this issue *via ad hoc* multivariate information domain approaches (Porta et al., 2017).

### Limitations

The main limitation of the present study is the small sample size and the lack of a control group of sedentary subjects. However, the assessment of the long-term effects of physical training in a group of athletes followed from their forties to fifties, an age particularly at risk for cardiac events, represents a strength of the study worth sharing. It must also be emphasized that the athletes of this study have changed the intensity of training over the years, a limitation inherent in the observational design of the study, which does not allow for considerations on the differences in the effects of strenuous or moderate exercise. *Ad hoc* studies are needed to elucidate the open questions.

## Conclusion

In the present study, we analyzed the QT variability and the HP and QT cardiac control complexity in a small group of middle-aged non-elite half-marathon runners evaluated at baseline and after 10 years.

Regular and moderate exercise over the years was beneficial for this group of athletes, as reflected by the decreased overall QT variability and the concomitant enhanced cardiac control complexity.

Taken together, these results point to a protective effect of physical exercise with respect to both ageing and cardiovascular risk, linked to a more flexible cardiac control with higher ability to interact and counterbalance external inputs, as it occurs in younger people.

## Data Availability

The raw data supporting the conclusions of this article will be made available by the authors, without undue reservation.
