# Supplementary material for: Improvement of Sympathovagal Balance by Regular Exercise May Counteract the Ageing Process. A Study by the Analysis of QT Variability
Source: Front Physiol. 2022 Apr 20;13:880250. doi: 10.3389/fphys.2022.880250 (PMC9065681; doi:10.3389/fphys.2022.880250)
Supplement: Supplementary file 1 [file Table1.DOCX]

**Supplementary 1. RR interval and QT interval variability parameters**

|  | **FU**  **(2018)** | | **B**  **(2008)** | |
| --- | --- | --- | --- | --- |
|  | **REST** | **STAND** | **REST** | **STAND** |
| µ_RR_, ms | 1069.67±155.87 | 856.07±141.02* | 930.31±194.69# | 765.90±192.34* |
| σ^2^_RR_, ms^2^ | 1727.56±717.73 | 1811.14±957.48 | 2711.12±1713.96 | 2577.85±1720.54 |
| LF_RR_, ms^2^ | 411.83±426.86 | 638.21±791.01 | 739.41±546.01# | 1053.37±939.61# |
| LF_nu,RR_, nu | 31.76±15.16 | 42.23±22.20* | 53.68±24.63# | 78.61±18.04*# |
| HF_RR_, ms^2^ | 691.62±220.91 | 434.79±199.77* | 630.43±761.49 | 358.30±685.99* |
| HF_nu,RR_, nu | 65.29±13.04 | 42.18±18.87* | 44.07±24.32# | 18.59±19.33*# |
| µ_QT_, ms | 373.96±36.27 | 338.78±38.63* | 348.81±39.06# | 326.63±39.20* |
| σ^2^_QT_, ms^2^ | 10.63±8.99 | 18.25±18.67 | 22.66±9.12# | 40.22±23.27*# |
| LF_QT_, ms^2^ | 2.06±1.89 | 4.35±5.05 | 3.94±4.00 | 8.30±7.51*# |
| HF_QT_, ms^2^ | 5.65±5.85 | 7.24±8.58 | 25.81±45.88 | 38.89±62.62 |

FU, follow-up; B, basal; REST, supine position; STAND, active standing; RR, RR interval; µ_RR_, RR mean; σ^2^_RR_, RR variance; LF, low frequency; LF_RR_, absolute power of RR in LF band; LF_RR,nu_, normalized power of RR in LF band; HF, high frequency; HF_RR_, absolute power of RR in HF band; HF_RR,nu_, normalized power of RR in HF band; QT, QT interval; µ_QT_, QT mean; σ^2^_QT_, QT variance; LF_QT_, absolute power of QT in LF band; HF_QT_, absolute power of QT in HF band. Data are presented as mean±standard deviation. * indicates p<0.05 REST vs STAND; # indicates p<0.05 B vs FU within the same experimental condition.
